# Supply, distribution and characteristics of international medical graduates in family medicine in the United States: a cross-sectional study

**DOI:** 10.1186/s12875-019-0933-8

**Published:** 2019-03-30

**Authors:** Robbert J. Duvivier, Elizabeth Wiley, John R. Boulet

**Affiliations:** 1 Foundation for Advancement of International Medical Education and Research, 3624 Market Street, Philadelphia, PA 19104-2685 USA; 2 School of Health Professions Education, Faculty of Health Medicine and Life Sciences, Maastricht University, Universiteitssingel 60, Maastricht, MD 6200 the Netherlands; 3 Parnassia Psychiatric Institute, Kiwistraat 43, The Hague, DH 2552 The Netherlands; 4 Johns Hopkins University Bloomberg School of Public Health, 615 N Wolfe St, Baltimore, MD 21205 USA; 5 Educational Commission for Foreign Medical Graduates, 3624 Market Street, Philadelphia, PA 19104-2685 USA

**Keywords:** Workforce, Family medicine, Primary care, International medical graduates, Medical school

## Abstract

**Background:**

To describe the supply, distribution, and characteristics of international medical graduates (IMGs) in family medicine who provide patient care in the U.S.

**Methods:**

A cross-sectional study design, using descriptive statistics on combined data from the Educational Commission for Foreign Medical Graduates and the American Medical Association, including medical school attended, country of medical school, and citizenship when entering medical school.

**Results:**

In total, 118,817 physicians in family medicine were identified, with IMGs representing 23.8% (*n* = 28,227) of the U.S. patient care workforce. Of all 9579 residents in family medicine, 36.0% (*n* = 3452) are IMGS. In total, 35.9% of IMGs attended medical school in the Caribbean (*n* = 10,136); 19.9% in South-Central Asia (*n* = 5607) and 9.1% in South-Eastern Asia (*n* = 2565). The most common countries of medical school training were Dominica, Mexico, and Sint Maarten. Of all IMGs in family medicine who attended medical school in the Caribbean, 74.5% were U.S. citizens. In total, 40.5% of all IMGs in family medicine held U.S. citizenship at entry to medical school. IMGs comprise almost 40% of the family medicine workforce in Florida, New Jersey and New York.

**Conclusions:**

IMGs play an important role in the U.S. family medicine workforce. Many IMGs are U.S. citizens who studied abroad and then returned to the U.S. for graduate training. Given the shortage of family physicians, and the large number of IMGs in graduate training programs, IMGs will continue to play a role in the U.S. physician workforce for some time to come. Many factors, including the supply of residency training positions, could eventually restrict the number of IMGs entering the U.S., including those contributing to family practice.

## Background

The number of international medical graduates (IMGs) practicing in the United States (U.S.) has steadily increased over the past fifty years, from 10% in 1963 [1] to currently about one-quarter of all physicians in active practice [2]. For decades, there has been a misalignment between the numbers of U.S. medical graduates (US-MGs) and Graduate Medical Education (GME) positions resulting in an excess of GME positions [3–5]. While requirements may vary by jurisdiction, all graduates, regardless of country of medical school training, must complete a minimum of 1 year of GME to obtain an unrestricted license to practice medicine. Given the availability of GME positions, and the need for physicians, the U.S. has historically relied on IMGs to fill residency positions [6, 7]. Recent data indicate that 24.6% of trainees in residency programs are IMGs [8] with even higher representation in the specialties (including subspecialties) of family medicine, internal medicine, neurology, nuclear medicine, pathology, paediatrics, and psychiatry. IMGs are also more likely to practice in counties with lower median incomes, to look after underserved populations, and to live and work in rural areas [9–12]. As a result, IMGs have been described as having “both gap-filling and safety-net roles” [13]. This is particularly relevant since US-MGs continue to show an overall preference for subspecialty careers [14–16], and proportionally fewer positions in family medicine are being filled by US-MGs. For example, whilst the total number of family medicine positions being offered has increased every year since 2008, and 10.5% between 2013 and 2017, the proportion of these positions filled by US-MGs stabilised around 44.0–45.3%. A large number of these positions are being filled by IMGs.

Against this background, knowing more about the supply, distribution and characteristics of IMGs in family medicine can help inform speciality-specific workforce policies. Previous research has addressed migration of IMGs in general [17, 18] or from specific regions [19, 20], and has focused on the geographical distribution of IMGs in particular specialties [13, 21, 22]. Few of these studies have focused on describing applicant characteristics by specialty and, more specifically, for family medicine. Morris et al. compared US-MGs to IMGs in family medicine and found significant differences in their professional profiles [23]. However, this study has several limitations, namely the currency and accuracy of the underlying data. To date, comprehensive analyses of the role of IMGs in family medicine in the U.S. have been limited. This study seeks to fill that gap by describing, based on the most current publicly available data, the characteristics of IMGs in family medicine who provide patient care in the U.S.Methods.

### Data sources

We used the 2017 American Medical Association Physician Masterfile (AMA Masterfile) to obtain information on physicians in the U.S. [24]. The AMA Masterfile includes data on all physicians who have met the requirements for recognition as a physician. A record is established when individuals enter medical schools accredited by the Liaison Committee on Medical Education (LCME) for US-MGs or, for IMGs, when they enter a residency training program accredited by the Accreditation Council for Graduate Medical Education (ACGME). Additional information is added from primary sources and surveys of the physicians listed in the Masterfile. We did not consider osteopathic residencies for the purpose of this study, as they are administered differently, require different medical licensing examinations, and are currently not open to IMGs. Data from the AMA Masterfile includes demographic information such as gender and birth country, as well as information on a physician’s training and career, such as year of graduation, practice specialty, geographical location of practice, type of practice, and present employment.

For this study, we merged the AMA Masterfile physician listings with data from the Educational Commission for Foreign Medical Graduates (ECFMG) using a unique identifier. ECFMG is the certifying body for all IMGs who wish to obtain a residency position in the U.S.; full certification requirements are described elsewhere [25]. To obtain an unrestricted license to practice medicine in any U.S. jurisdiction, an IMG must complete at least 2 years, and often 3 years depending on the licensing authority, of residency training. Data from ECFMG used in this study include country of citizenship at the time of entry into medical school, country of medical school training, and medical school attended.

### Variables

We used the demographic information available in our combined dataset of AMA and ECFMG records. The AMA Masterfile contains details on physicians who currently practice in the U.S., including their specialty, type of practice and major professional activity. With respect to specialty, the AMA Masterfile contains over 200 self-designated practice specialties. We selected only those individuals who indicated a family medicine specialty or subspecialty. This included Family Medicine, Family Practice, Adolescent Medicine for Family Practice, Family Medicine/Psychiatry, General Practice, Sports Medicine (Family Medicine), Emergency Medicine/Family Medicine, Family Medicine/ Preventive Medicine, Geriatric Medicine (Family Medicine), Hospice & Palliative Medicine (Family Medicine), and Internal Medicine/ Family Medicine.

In terms of the major professional activity, we included all physicians involved in patient care activities whose primary self-designated practice specialty was one of the family medicine designations listed above. We included residents, full-time hospital staff, physicians in office-based practice and locum tenens. We excluded individuals whose self-designated major professional activity was research, administration, medical teaching, or who were listed as inactive. There was no adjustment made for full-time equivalents, since that information is not consistently available.

Other information derived from the AMA Masterfile included the type of employment (solo practice, state hospital, etc.), location of practice (by postal code), and type of physician (IMG, U.S. MD or U.S. DO (osteopathic physician). In the U.S., graduates from an osteopathic medical school can specialize in family medicine and work as family physicians. They have comparable training and certification requirements as MD family physicians. Since they play an important role in the delivery of care in family medicine, we included DOs in our analyses.

For IMGs, we obtained additional information from the ECFMG records. As part of the certification process, ECFMG collects demographic data including citizenship at the time of entry into medical school and country of medical school. In line with common practice [26], an IMG was defined as an individual who graduated from a medical school located outside of Canada or the U.S., regardless of citizenship. We used information about citizenship at the time of entry into medical school to classify IMGs as either U.S. citizens who graduated from a medical school located outside of Canada or the U.S. (US-IMGs), or non-U.S. citizen IMGs (non-US-IMGs).

### Analysis

Descriptive statistics were used to show the distribution and characteristics of physicians in family medicine who provide patient care in the U.S. Where appropriate, we made comparisons between IMGs and US-MGs, including both MDs and DOs. We did not apply any inferential statistics since the study group includes the whole population of practicing physicians in family medicine designated specialty categories.

## Results

The 2017 AMA Masterfile, which primarily reflects the status of the workforce at the end of 2016, includes 907,731 physicians who are in the U.S. and involved in patient care activities, including full-time hospital staff, individuals in office-based practice, residents, and doctors doing locum tenens. For those physicians involved in patient care for whom a self-designated practice specialty was available (96.1%), including IMGs, MDs and DOs, there are 118,817 in family medicine-designated practice specialties. Overall, physicians in family medicine make up 13.1% of the entire physician patient care workforce. Self-designated practice specialties include Family Medicine (*n* = 98,929, 83.3%), Family Practice (*n* = 9095, 7.7%), General Practice (*n* = 7634, 6.4%), Sports Medicine (Family Medicine)(*n* = 1849, 1.6%), Geriatric Medicine (Family Medicine) (*n* = 954, 0.8%), Family Medicine/ Psychiatry (*n* = 128, 0.1%), Emergency Medicine/ Family Medicine (*n* = 71, 0.1%), Internal Medicine/ Family Medicine (*n* = 69, 0.1%), Family Medicine/ Preventive Medicine (*n* = 51, <.1%), Hospice & Palliative Medicine (Family Medicine)(*n* = 28, <.1%), Adolescent Medicine for Family Practice (*n* = 9, <.1%).

Those providing patient care (full-time hospital staff, physicians in office-based practice, residents, and locum tenens) whose self-designated specialty is family medicine, were most commonly in office-based practice (*n* = 98,233; 82.7%), full-time hospital staff (*n* = 10,708; 9.0%), residents (*n* = 9579; 8.1%) and locum tenens (*n* = 297; 0.2%).

A breakdown of the family medicine workforce (i.e. those involved in patient-care activities) is provided in Table 1.

**Table 1:** Family Medicine Workforce (in Patient-Care Activities) in 2017

Type	N	%
U.S. Citizen IMG (US-IMG)	11,259	9.5
Non-U.S. Citizen IMG (non-US-IMG)	16,528	13.9
Unknown IMG	440	0.4
All IMG	28,227	23.8
U.S. MD	71,473	60.2
U.S. DO	19,117	16.1
All Family Medicine (in patient care)	118,817	

International Medical Graduates (*n* = 28,227) represent 23.8% of the family medicine workforce who provide patient care. For all other self-designated specialties, excluding physicians not involved in patient care, IMGs (*n* = 218,059) represent 24.0% of the total (*n* = 907,731) U.S. patient care workforce. Eliminating the IMGs for whom citizenship information at time of entry to medical school was not available (*n* = 440), US-IMGs make up 40.5% of the internationally-educated family medicine workforce.

The majority of physicians in family medicine are male (60.3%), with a similar breakdown for IMGs (57.5%) and U.S. MDs and DOs (61.1%). Compared with U.S. MDs and DOs practicing in family medicine (mean age = 50.7 years; standard deviation [SD] = 12.3), practicing IMGs, on average, are a little younger (mean = 49.2 years; SD = 13.7).

Based on employment data from the AMA Physician Masterfile, IMGs practicing in family medicine are less likely to work in a group practice (*n* = 9261; 32.8%) than U.S. MDs or DOs (*n* = 44,003; 48.6%). IMGs in family medicine are more likely to be in self-employed solo practice (*n* = 4644; 16.5%) than U.S. MDs or DOs (*n* = 12,006; 13.3%).

### IMGs in family medicine

Of the 28,227 IMGs in family medicine, 3452 are residents (36.0% of all 9579 residents in family medicine); 22,361 are in office-based practice (22.8% of all 98,233 physicians in family medicine in office-based practice); and 2344 are full-time hospital staff (21.9% of all 10,708 physicians in family medicine who were full-time hospital staff).

Country of medical school training was available for 28,104 (99.6%) of all IMGs currently providing patient care as family medicine practitioners. The top 20 (of 150) countries are shown in Table 2. More than one-third of IMGs attended medical school in the Caribbean^1^ (*n* = 10,136; 35.9%), with over one quarter graduating from a medical school in South-Central Asia^2^ (*n* = 5607; 19.9%) or South-Eastern Asia^3^ (*n* = 2565; 9.1%).

**Table 2:** Medical School Country for IMGs in Patient Care Activities in Family Medicine (top 20) in 2017

Medical School Country	N	%
India	3867	13.76
Dominica	2623	9.33
Mexico	2302	8.19
Philippines	2198	7.82
Sint Maarten	1600	5.69
Grenada	1589	5.65
Dominican Republic	1414	5.03
Pakistan	1049	3.73
Saint Kitts and Nevis	575	2.05
Poland	510	1.81
Cuba	506	1.80
Antigua and Barbuda	480	1.71
saba	475	1.69
Cayman Islands	448	1.59
Nigeria	434	1.54
Egypt	424	1.51
Spain	376	1.34
China	372	1.32
Iran	325	1.16
Colombia	312	1.11

Citizenship at time of medical school was available for 27,769 (98.4%) of the IMGs in family medicine. The countries with more than 100 citizens (at entry to medical school) in the IMG cohort are presented in Table 3.

**Table 3:** Citizenship at Entry to Medical School for IMGs in Patient Care Activities in Family Medicine in 2017

Country of citizenship	Frequency	Percent
United States of America	11,241	40.48
India	3855	13.88
Philippines	1806	6.50
Canada	1107	3.99
Pakistan	1046	3.77
Cuba	513	1.85
Nigeria	500	1.80
Iran	432	1.56
China	417	1.50
Egypt	403	1.45
Mexico	348	1.25
Colombia	304	1.09
United Kingdom	279	1.00
Ussr	276	0.99
Vietnam	234	0.84
Poland	222	0.80
Korea	210	0.76
Syria	185	0.67
Haiti	164	0.59
Iraq	164	0.59
Dominican Republic	162	0.58
Peru	161	0.58
Romania	159	0.57
Bangladesh	148	0.53
Russia	135	0.49
Taiwan	127	0.46
Nicaragua	124	0.45
South Africa	111	0.40
Germany	107	0.39
Myanmar (Burma)	106	0.38
Argentina	103	0.37

Analysis at country level showed that for the 11,241 US-IMGs in our dataset (39.8% of all IMGs in family medicine), the most common countries of medical school training were Dominica (*n* = 2213; 19.7%), followed by Mexico (*n* = 1682; 15.0%) and Sint Maarten (*n* = 1397; 12.4%). Of all the practicing IMGs in family medicine who attended medical school in the Caribbean (*n* = 10,316), 7569 (74.5%) were U.S. citizens at time of entry into medical school. A large number of IMG family practitioners who attended Caribbean medical schools were Canadian citizens (*n* = 919, 9.1%). Even for other countries in the top 20 (based on country of medical school), many students were U.S. citizens. For example, of the 3867 IMG family physicians who attended medical school in India, 157 (4.1%) were U.S. citizens at entry to medical school.

Information on the medical school of primary medical degree was available for 27,845 (98.6%) of all IMGs in family medicine. Table 4 provides the top 15 provider schools for IMGs in family medicine. Of these, 9 are located in the Caribbean, 3 in the Philippines, 2 in Mexico and one in Pakistan. Together, they account for just over 40% of all IMGs in family medicine.

**Table 4:** Medical School Attended for Practicing IMGs in Family Medicine (top 15)

Medical School	Country	Number	Percent	Aggregate Percentage of all IMGs in Family Medicine
Ross University School of Medicine	Dominica	2611	9.38	
St. George’s University School of Medicine	Grenada	1584	5.69	15.07
Universidad Autónoma de Guadalajara Facultad de Medicina Guadalajara	Mexico	1410	5.06	20.13
American University of the Caribbean School of Medicine	Sint Maarten	1388	4.98	25.11
University of Santo Tomas Faculty of Medicine and Surgery	Philippines	706	2.54	27.65
Universidad Central del Este (UCE) Facultad de Medicina	Dominican Republic	563	2.02	29.67
Saba University School of Medicine	Saba	475	1.71	31.38
St. Matthew’s University School of Medicine (Grand Cayman)	Cayman Islands	448	1.61	32.99
American University of Antigua College of Medicine	Antigua and Barbuda	436	1.57	34.56
University of the East/Ramon Magsaysay Memorial Medical Center College ofMedicine	Philippines	357	1.28	35.84
Universidad de Ciencias Médicas de la Habana	Cuba	314	1.13	36.97
Far Eastern University Institute of Medicine, Nicanor Reyes Medical Foundation	Philippines	289	1.04	38.01
Medical University of the Americas (Nevis)	Saint Kitts and Nevis	272	0.98	38.99
Dow Medical College	Pakistan	266	0.96	39.95
American University of Integrative Sciences, St. Maarten School of Medicine	Barbados	212	0.76	40.71
Total			40.71	

International medical graduates are practicing family medicine in all 50 States. Table 5 shows the number of IMGs in family medicine by state (top 10). Many of these states have large proportions of IMGs across all specialties (right-hand column, for reference), although in some states IMGs in family medicine are practicing in even greater proportions. In Florida, New Jersey and New York, IMGs comprise almost 40% of the family medicine workforce.

**Table 5:** Top 10 States with Practicing IMGs in Family Medicine in 2017

State	N IMGs in FM	% of all IMGs in FM	All FM	% IMG FM	All Physicians in Patient Care	All IMGs in Patient Care	% IMG in Patient Care
CALIFORNIA	3930	13.97	13,590	28.9	104,996	26,808	25.5
FLORIDA	2685	9.55	6731	39.9	53,953	19,005	35.2
TEXAS	2299	8.17	8725	26.3	62,622	15,346	24.5
NEW YORK	1886	6.71	4810	39.2	73,724	26,808	36.4
ILLINOIS	1753	6.23	4802	36.5	37,062	11,000	29.7
MICHIGAN	1232	4.38	4228	29.1	30,324	8728	28.8
PENNSYLVANIA	1045	3.72	5294	19.7	41,927	9731	23.2
GEORGIA	862	3.07	2978	28.9	23,078	5041	21.8
NEW JERSEY	836	2.97	2184	38.2	28,262	11,264	39.8
OHIO	814	2.89	4335	18.8	34,999	8201	23.4

## Discussion

International medical graduates play an important role in the U.S. family medicine workforce. Our analysis shows that IMGs account for 23.8% of the family medicine workforce, in line with earlier published work in other specialties (e.g. 13). Many states are highly reliant on IMGs to fill their workforce needs. Our detailed analysis of the IMG family medicine workforce indicates great diversity with respect to citizenship, country of medical school training, and various practice-based demographics. One-third of IMGs attended medical school in the Caribbean confirming that this region has, at least historically, been a major supplier of physicians to the U.S. [27]. Over one quarter graduated from medical school in South-Central/South-Eastern Asia, which is a reflection of the wider migration of IMGs from this region to the U.S. [28]. More importantly, over one-third of family medicine residents are IMGs. Provided that they complete training and eventually practice in the U.S., the contribution of IMGs to the practicing FM workforce is likely to increase, at least in the short-term.

We further established that the country of medical degree is not a good proxy for, or indication of, IMG nationality; 40.5% of IMGs in family medicine held US citizenship at entry to medical school. These US-IMGs most frequently attended medical school in Mexico and the Caribbean. Nearly three-quarters of all physicians in family medicine who graduated from a Caribbean medical school were US citizens with a further 9.1% being Canadian. These results resonate with a pattern of migration that was identified in a different population, namely African-trained IMGs [20]. The authors found that there are a number of US citizens who had moved away from the U.S. to pursue a medical degree in an African country, only to return after graduation for U.S.-based GME. While the IMGs entering family medicine residencies and practicing in the US are a diverse group with many, by virtue of being U.S. citizens and, likely having been raised in the U.S., already being acculturated to the U.S. healthcare system. This can be beneficial in that resources required to orient these individuals to the idiosyncrasies of patient care in the U.S. are reduced. However, from a patient care perspective, a more diverse pool of physicians in terms of ethnicity and languages spoken may better align with the needs of the U.S. patient population [18].

Our findings have a number of workforce implications. While U.S. citizens studying medicine abroad are classified as IMGs, from a workforce planning perspective, they are typically counted in estimates of “brain drain”. However, most U.S. citizens who leave the U.S. for medical education have no intention of staying abroad. Therefore, their contribution to international health workforce disparities, or U.S. debt attributable to the subsidy of public education, is minimal. The workforce disparity issue is particularly relevant for countries with a disproportionate number of medical schools compared to the local labour market, as is true in the Caribbean [28–30]. Although many of the medical schools in the Caribbean are for-profit, and cater to IMGs, the sustainability of “offshore” education of U.S. citizens, could be questioned given the increase in U.S. MD and DO enrolment and the lack of additional public funding for U.S. GME positions,. Furthermore, countries where supplier schools are located might provide public funding to train doctors who are subsequently moving to the U.S. We acknowledge the debate around ‘poaching’ of physicians by several countries, including the US [31, 32]. However, U.S. citizens who go abroad for medical education do not contribute to “brain drain”.

The current U.S. family physician workforce is highly dependent on IMGs. This may change in the future. Although projections by the Association for American Medical Colleges suggest physician shortages in the upcoming decades, there is a downward trend in the number of IMGs applying for the residency Match in recent years [33]. More important, the number of graduates from U.S. medical schools may eventually surpass the number of available residency positions [34, 35]. The total number of US-MGs has been increasing as class sizes of existing medical programs have expanded [36, 37]. Moreover, new medical schools will begin to graduate their incoming classes; almost all of these graduates will be competing for GME positions. With 18 new U.S. medical schools established over the last 10 years and 9 in the last 5 years [38], there will be approximately 7000 additional US-MGs every year [39]. If these individuals seek GME positions, the availability of graduate training slots for IMGs is likely to decrease. In the U.S., the number of residency positions has remained relatively unchanged, increasing by approximately 1.6% per year [40]. This is largely the result of the “cap” on federal financing of GME positions through Medicare, or “slots” [34]. Although there are still far more positions available than US-MGs, competition for these positions is increasing [41, 42]. Because US-MGs tend to remain in their home country for specialty training, and many programs will continue to give priority to US-MGs over IMGs, the number of IMGs who will be able to pursue specialty training in the U.S. is expected to decrease [4, 5, 35]. Furthermore, recent U.S. immigration policy changes may make it more difficult, at least for non-U.S. citizen IMGs, to obtain visas to travel to, or to work in the U.S., thus making them less likely to be selected into residency training programs [43, 44]. Given these issues, the U.S. dependency on IMGs, including family physicians, is likely to decrease. However, based on current licensure rules, the estimated shortage in primary care physicians of 7300 to 43,100 by 2030 [45] will require additional GME positions. Barring an influx of US-MDs or DOs seeking primary care specialties, at least some internationally-trained physicians will still be needed to fill the workforce gap [40].

The prevalence of US-IMGs in family medicine may raise concerns regarding the quality of primary care. Although there are many factors that determine the quality of a physician, including undergraduate medical education, it is unclear whether current certification and licensure requirements provide sufficient safeguards to ensure that IMGs deliver high-quality care. There is a growing body of literature suggesting that the quality of care provided by US-IMGs may be inferior to that of US-MGs and indeed other IMGs. The available studies show that US-IMG scores on United States Medical Licensing Examinations vary considerably [46], they are less likely to be specialty Board certified [47, 48] and, at least for some patient conditions, provide less adequate care [49–51]. If these findings amongst all US-IMGs generalize to Family Medicine, and there are more US-IMGs going into the specialty, the overall quality of patient care could suffer. This is particularly relevant given the number of –US-IMGs in Family Medicine, many of whom graduated from medical schools in the Caribbean where, historically, there has been considerable variability in performance [27, 46, 47]. Finally, given that IMGs are less likely to work in group practices, and fewer family physicians overall are working as solo practitioners [52], access to, and quality of care, could be impacted.

### Limitations

There are a number of limitations of our study. First, the underlying data set, the AMA Masterfile, has been reported to under/over-represent different specialties and practice settings [53, 54]. Second, some of the variables used in the analyses were self-reported (e.g., primary self-designated specialty, citizenship) and may be subject to error. It seems unlikely, however, that individuals would purposefully distort their responses. Third, our study results were based on a cross-sectional analysis of physician practice data. While beyond the scope of this investigation, the longitudinal analysis of ECFMG application trends and IMG contributions to family medicine would clearly improve any projections concerning the future composition of the US workforce.

## Conclusion

Our study offers a closer look at the characteristics of IMGs in the family medicine workforce. We have discussed the implications of our findings in the context of current conditions, namely that the future workforce physician shortages are unlikely to be alleviated entirely by domestically educated physicians.

## Abbreviations

ACGME: Accreditation Council for Graduate Medical Education
AMA: American Medical Association
ECFMG: Educational Commission for Foreign Medical Graduates
GME: Graduate Medical Education
IMG: International Medical Graduate
LCME: Liaison Committee on Medical Education
US-MG: United States Medical Graduate

## Footnotes

1 Anguilla, Antigua and Barbuda, Aruba, Bahamas, Barbados, Bonaire, Sint Eustatius, Saba, British Virgin Islands, Cayman Islands, Cuba, Curaçao, Dominica, Dominican Republic, Grenada, Guadeloupe, Haiti, Jamaica, Martinique, Montserrat, Puerto Rico, Saint-Barthélemy, Saint Kitts and Nevis, Saint Lucia, Saint Martin (French part), Saint Vincent and the Grenadines, Sint Maarten (Dutch part), Trinidad and Tobago, Turks and Caicos Islands, United States Virgin Islands.

2 Afghanistan, Bangladesh, Bhutan, India, Nepal, Maldives, Pakistan and Sri Lanka.

3 Brunei, Cambodia, East Timor, Indonesia, Laos, Malaysia, Myanmar (Burma), Philippines, Singapore, Thailand and Vietnam.

## References

[1] Salsberg ES, Forte GJ (2002). Trends in the physician workforce, 1980–2000. Health Aff (Millwood).

[2] ECFMG | Annual Report. Available from: https://www.ecfmg.org/resources/data-certification.html. [cited 2017 Mar 7].

[3] Iglehart JK (2013). The residency mismatch. N Engl J Med.

[4] Boulet JR, Norcini JJ, Whelan GP, Hallock JA, Seeling SS (2006). The international medical graduate pipeline: recent trends in certification and residency training. Health Aff (Millwood)..

[5] Whelan GP, Gary NE, Kostis J, Boulet JR, Hallock JA (2002). The changing pool of international medical graduates seeking certification training in US graduate medical education programs. JAMA.

[6] Hagopian A, Thompson MJ, Kaltenbach E, Hart LG (2003). Health departments’ use of international medical graduates in physician shortage areas. Health Aff (Millwood)..

[7] Hart LG, Skillman SM, Fordyce M, Thompson M, Hagopian A, Konrad TR (2007). International medical graduate physicians in the United States: changes since 1981. Health Aff (Millwood)..

[8] Brotherton SE, Etzel SI (2016). Graduate medical education, 2015-2016. JAMA..

[9] Mick SS, Lee S-YD, Wodchis WP (2000). Variations in geographical distribution of foreign and domestically trained physicians in the United States:‘safety nets’ or ‘surplus exacerbation’?. Soc Sci Med.

[10] Baer LD, Ricketts TC, Konrad TR, Mick SS (1998). Do international medical graduates reduce rural physician shortages?. Med Care.

[11] Whitcomb ME, Miller RS (1995). Participation of international medical graduates in graduate medical education and hospital care for the poor. JAMA..

[12] Thompson MJ, Hagopian A, Fordyce M, Hart LG (2009). Do international medical graduates (IMGs)“fill the gap” in rural primary care in the United States? A national study. J Rural Health.

[13] Boulet JR, Cassimatis EG, Opalek A (2012). The role of international medical graduate psychiatrists in the United States healthcare system. Acad Psychiatry.

[14] Rosenblatt RA, Chen FM, Lishner DM, Doescher MP (2015). The future of family medicine and implications for rural primary care physician supply. WWAMI Rural Health Research Center. final report.

[15] Biggs WS, Crosley PW, Kozakowski SM (2013). Results of the 2013 National Resident Matching Program®. Fam Med.

[16] Bieck AD, Biggs WS, Crosley PW, Kozakowski SM (2012). Results of the 2012 National Resident Matching Program: family medicine. Fam Med.

[17] Mullan F (2005). The metrics of the physician brain drain. N Engl J Med.

[18] Norcini JJ, van Zanten M, Boulet JR (2008). The contribution of international medical graduates to diversity in the US physician workforce: graduate medical education. J Health Care Poor Underserved.

[19] Tekian A, Boulet J (2015). A longitudinal study of the characteristics and performances of medical students and graduates from the Arab countries. BMC Med Educ.

[20] Duvivier RJ, Burch VC, Boulet JR (2017). A comparison of physician emigration from Africa to the United States of America between 2005 and 2015. Hum Resour Health.

[21] Halpern JA, Al Awamlh BAH, Mittal S, Shoag JE, Hu JC, Lee RK (2016). International medical graduate training in urology: are we missing an opportunity?. Urology.

[22] Schenarts PJ, Love KM, Agle SC, Haisch CE (2008). Comparison of surgical residency applicants from US medical schools with US-born and foreign-born international medical school graduates. J Surg Educ.

[23] Morris AL, Phillips RL, Fryer GE, Green LA, Mullan F (2006). International medical graduates in family medicine in the United States of America: an exploration of professional characteristics and attitudes. Hum Resour Health.

[24] AMA Physician Masterfile. Available from: https://www.ama-assn.org/practice-management/masterfile/ama-physician-masterfile. [cited 2015 Aug 4].

[25] ECFMG | Certification. Available from: http://www.ecfmg.org/certification/. [cited 2017 Mar 8].

[26] ECFMG | Definition of an IMG. Available from: http://www.ecfmg.org/certification/definition-img.html. [cited 2017 Mar 8].

[27] van Zanten M, Boulet JR. Medical Education in the Caribbean: Quantifying the Contribution of Caribbean-Educated Physicians to the Primary Care Workforce in the United States. Acad Med. 2013;88(2):276–81. 10.1097/ACM.0b013e31827c6cd3 23269307

[28] McAvinue MB, Boulet JR, Kelly WC, Seeling SS, Opalek A (2005). US citizens who graduated from medical schools outside the United States and Canada and received certification from the educational Commission for Foreign Medical Graduates, 1983–2002. Acad Med.

[29] Eckhert NL, van Zanten M (2015). US-citizen international medical graduates—a boon for the workforce?. N Engl J Med.

[30] Duvivier RJ, Boulet JR, Opalek A, Zanten M, Norcini J (2014). Overview of the world’s medical schools: an update. Med Educ.

[31] Hagopian A (2007). Recruiting primary care physicians from abroad: is poaching from low-income countries morally defensible?. Ann Fam Med.

[32] Snyder J (2009). Is health worker migration a case of poaching?. Am J Bioeth.

[33] National Resident Matching Program (2017). Main match results and data 2017.

[34] Iglehart JK (2011). The uncertain future of Medicare and graduate medical education. N Engl J Med.

[35] Traverso G, McMahon GT (2012). Residency training and international medical graduates: coming to America no more. JAMA..

[36] Table B-1: Total Enrollment by U.S. Medical School and Sex - Enrollment, Graduates, and MD/PhD Data - FACTS: Applicants, Matriculants, Enrollment, Graduates, MD/PhD, and Residency Applicants Data - Data and Analysis - AAMC. Available from: https://www.aamc.org/data/facts/enrollmentgraduate/158808/total-enrollment-by-medical-school-by-sex.html. [cited 2017 Mar 8].

[37] Barzansky B, Etzel SI (2016). Medical Schools in the United States, 2015–2016. JAMA..

[38] Association of American Medical Colleges. A Snapshot of the New and Developing Medical Schools in the US and Canada.pdf [Internet]. Available from: https://store.aamc.org/a-snapshot-of-the-new-and-developing-medical-schools-in-the-u-s-and-canada-pdf.html. [cited 2015 Aug 4].

[39] Association of American Medical Colleges. Physician Supply and Demand Through 2025: Key Findings. Available from: https://www.aamc.org/download/153160/data/physician_shortages_to_worsen_without_increases_in_residency_tr.pdf. [cited 2015 Aug 4].

[40] Mullan F, Salsberg E, Weider K (2015). Why a GME Squeeze Is Unlikely. N Engl J Med..

[41] Jolly P, Erikson C, Garrison G (2013). US graduate medical education and physician specialty choice. Acad Med..

[42] Desbiens NA, Vidaillet HJ (2010). Discrimination against international medical graduates in the United States residency program selection process. BMC Med Educ..

[43] Masri A, Senussi MH (2017). Trump’s Executive Order on Immigration—Detrimental Effects on Medical Training and Health Care. N Engl J Med..

[44] Duvivier RJ, Abdou MH, Ishak RS, Wiley E, Alwan MB (2017). Implications of a travel ban on US medical education and training. The Lancet..

[45] IHS Markit Ltd. 2018 Update: The Complexities of Physician Supply and Demand: Projections From 2016 to 2030: Final Report. Washington, DC: Association of American Medical Colleges; 2018.

[46] van Zanten M, Boulet JR (2008). Medical education in the Caribbean: variability in medical school programs and performance of students. Acad Med..

[47] Norcini JJ, McKinley DW, Boulet JR, Anderson MB (2006). Educational Commission for Foreign Medical Graduates certification and specialty board certification among graduates of the Caribbean medical schools. Acad Med..

[48] Boulet JR, Swanson DB, Cooper RA, Norcini JJ, McKinley DW (2006). A comparison of the characteristics and examination performances of US and non-US citizen international medical graduates who sought Educational Commission for Foreign Medical Graduates certification: 1995–2004. Acad Med..

[49] Norcini JJ, Boulet JR, Dauphinee WD, Opalek A, Krantz ID, Anderson ST (2010). Evaluating the quality of care provided by graduates of international medical schools. Health Aff (Millwood)..

[50] Tamblyn R, Abrahamowicz M, Dauphinee WD, Hanley JA, Norcini J, Girard N (2002). Association between licensure examination scores and practice in primary care. JAMA..

[51] Norcini JJ, Kimball HR, Lipner RS (2000). Certification and specialization: do they matter in the outcome of acute myocardial infarction?. Acad Med..

[52] Peterson LE, Baxley E, Jaén CR, Phillips RL (2015). Fewer family physicians are in solo practices. J Am Board Fam Med..

[53] Williams PT, Whitcomb M, Kessler J (1996). Quality of the family physician component of the AMA Masterfile. J Am Board Fam Pract..

[54] Henderson M (2015). Assessing the Accuracy of Three National Physician Sampling Frames. J Gen Intern Med.

